# Training and Transfer Effects of Combining Inhibitory Control Training With Transcutaneous Vagus Nerve Stimulation in Healthy Adults

**DOI:** 10.3389/fpsyg.2022.858938

**Published:** 2022-04-18

**Authors:** Chunchen Wang, Xinsheng Cao, Zhijun Gao, Yang Liu, Zhihong Wen

**Affiliations:** Department of Aerospace Medicine, Fourth Military Medical University, Xi’an, China

**Keywords:** inhibitory control training, tVNS, training effect, near-transfer effect, far-transfer effect

## Abstract

Inhibitory control training (ICT) is a promising method to improve individual performance of inhibitory control (IC). Recent studies have suggested transcutaneous vagus nerve stimulation (tVNS) as a novel approach to affect cognitive function owing to its ability to modulate the locus coeruleus-noradrenaline system. To examine the synergistic effects of combining ICT with tVNS, 58 young males in college were randomly assigned to four groups: ICT + tVNS, ICT + sham tVNS, sham ICT + tVNS, and sham ICT + sham tVNS. Participants were instructed to complete three sessions that comprised pre-training tests, a training session, and post-training tests sequentially. Results showed that the ICT + tVNS group significantly improved training and near-transfer effects on the stop-signal and Go/No-go tasks, and these effects were larger than those of the other groups. However, none of the groups exhibited the far-transfer effect on the color-word Stroop task. These results suggest that tVNS augments the intervention effects of training and similar inhibition tasks to achieve the synergistic effect; however, it does not modulate the effects of non-training tasks and obtain the far-transfer effect. ICT combined with tVNS may be a valuable intervention for improving IC in healthy individuals in certain industries and offers novel research ideas for using tVNS for cognitive improvement.

## Introduction

Inhibitory control (IC) is a high-level cognitive executive function (Miyake et al., 2000; Verbruggen and Logan, 2008; Diamond, 2013) that plays a key role in the cognitive skills and mental well-being of daily human life. IC is defined as the active suppression of interference or prepotent responses and consists of response inhibition and conflict inhibition. Poor IC in healthy individuals is inversely correlated with the performance of important tasks, such as military actions (Biggs et al., 2015; Brunyé et al., 2020) and driving and flighting in traffic accidents (Mäntylä et al., 2009; Dismukes et al., 2018; Hamilton et al., 2019; Yan et al., 2021). Furthermore, deficits in IC are commonly observed in patients with neurological disorders, such as attention deficit hyperactivity disorder (ADHD) (Re et al., 2015; Scionti et al., 2019; Einziger et al., 2021), autism spectrum disorder (Yuk et al., 2020), and mild cognitive impairment (Rabi et al., 2020; Yuk et al., 2020). Therefore, IC improvement are of great significance for individuals engaged in certain occupations and patients with neurological disorders.

In recent years, targeted cognitive training and non-invasive brain stimulation have become popular approaches for improving and enhancing individual IC. IC training (ICT) was proposed, which involves cognitive training using IC paradigms (e.g., Go/No-go, stop-signal task (SST), and Stroop tasks) to improve performance on training tasks (called the training effect) (Hofmann and Forster, 2019; Zhao and Jia, 2019) and non-training tasks with similar inhibitory function components (called the near-transfer effect) (Kassai et al., 2019), which is attributed to the activation of specific brain regions that are closely related to IC during the training process (Thorndike and Woodworth, 1901; Blacker et al., 2018). However, the effects of IC improvement are too weak to be observed in healthy individuals with high baseline cognitive functions (Burki et al., 2014; Zhao and Jia, 2019). Moreover, inconsistent findings have indicated that the benefits of cognitive training of IC should not transfer to tasks that refer to other components of inhibitory function, different from training tasks (called the far-transfer effect) (Kassai et al., 2019; Sala and Gobet, 2019). For instance, the performance of conflict inhibition should not be enhanced by cognitive training of response inhibition.

Another new approach for improving IC is non-invasive brain stimulation, such as transcutaneous vagus nerve stimulation (tVNS), which is a novel non-invasive intervention for modulating nervous system function by applying direct-current pulse electrical stimulation on the ear branch of the vagus nerve (Ventureyra, 2000). Recent studies have shown that tVNS activates the locus coeruleus-noradrenergic (LC-NA) system (Warren et al., 2019; Ricci et al., 2020; Sharon et al., 2021). The LC-NA system modulates and improves cognitive functions, such as IC. Several studies focusing on IC improvement using tVNS have found no significant differences compared to sham tVNS in the behavioral data of Go/No-go and Simon tests. However, it has been reported that tVNS enhances the performance of electrophysiological markers of conflict inhibition, such as the N2 and P3 components of event-related potentials (ERPs) (Fischer et al., 2018; Jongkees et al., 2018; Pihlaja et al., 2020) and reduced conflict costs on behavioral performance in go trials. Therefore, tVNS may improve the efficiency of the neuromodulation process in IC and enable better IC performance to be achieved with fewer neural resources (Van Leusden et al., 2015; Brunyé et al., 2020).

Given the limitations and varied characteristics of targeted cognitive training and non-invasive brain stimulation, combining these approaches may be a novel and effective approach for improving IC in individuals because it may produce synergistic effects and augment training and transfer effects by improving the method of targeted cognitive training (Elmasry et al., 2015; Oldrati et al., 2018; Mannarelli et al., 2020).

Therefore, the present study aimed to investigate whether combining ICT with tVNS produces synergistic effects on improving IC. We hypothesized that the modulation of the LC-NA pathway *via* tVNS would augment the training, near-transfer, and far-transfer effects of ICT using a set of IC tests.

## Materials and Methods

### Participants

Sixty healthy young males were recruited from the fourth military medical university using recruitment leaflets. Two participants were excluded because of study withdrawal after the pre-training test. Thus, our study included 58 participants who were college undergraduates, right-handed, and aged 18–21 years (mean age 19.5 ± 0.7 years) who had not participated in similar research previously. The sample size was predetermined by G*Power software (latest ver. 3.1.9.7.2; Heinrich-Heine-Universität Düsseldorf, Düsseldorf, Germany) (Kang, 2021). Based on a medium effect size (Cohen’s *d* = 0.25) and a significant effect (alpha = 0.05, Power = 0.80, ANOVA: repeated measures, within-between interaction, four groups and two measures), total sample size was 48 (12 participants per group). Sixty participants (15 participants per group) were recruited to avoid the potential risks that participants dropped out of the experiment and invalid experimental data appeared in participants. According to the actual sample size, the actual *post hoc* power by G*Power software was 0.88. Other exclusion criteria included color blindness, history of any psychological or neurological disorders, brain trauma or surgery, heart-related diseases, and adult ADHD assessed using the adult ADHD self-report scale (Kessler et al., 2005). This research was approved by the medical ethics committee of the Air Force Medical University (NO.KY20213079-1), and all procedures were carried out in accordance with the Declaration of Helsinki. Participants provided informed written consent prior to participating in the experiment and received 100 RMB/h as compensation for completing the experimental tasks efficiently.

### Design

**FIGURE 1:**
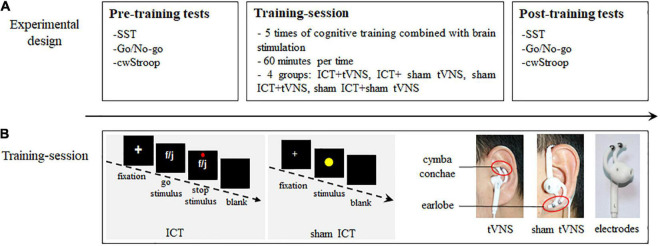
Research schedule. **(A)** Experimental design: the three sessions of pre- and post-training tests and training-session. **(B)** Training-session: (1) The SST framework in ICT; (2) The SRT framework in sham ICT; (3) Position of tVNS on the cymba conche of the left ear; (4) Position of sham tVNS on the earlobe of the left ear; (5) The Ag/AgCl electrodes.

Participants were allocated to one of four groups using a randomized single-blinded method: ICT + tVNS (*n* = 14, mean age 19.50 ± 0.65 years), ICT + sham tVNS (*n* = 15, mean age 19.53 ± 0.83 years), sham ICT + tVNS (*n* = 15, mean age 19.33 ± 0.72 years), and sham ICT + sham tVNS (*n* = 14, mean age 19.64 ± 0.74 years). The research schedule for each participant lasted for around 2 weeks and comprised three sessions (Figure 1): pre-training tests, training session, and post-training tests sequentially. The pre-training tests were completed 1–2 days before the training session. The post-training tests were completed 1–2 days after the training session. The training session schedule consisted of five sessions of combined simultaneous ICT and tVNS. The training frequency was once a day, and each training session lasted approximately 60 min, where participants were required to complete four sets of combined simultaneous ICT and tVNS intervention. Each set of ICT comprised 240 trials, and a 5 min break was given between sets. The tVNS applied during the ICT also included five sessions. The frequency of tVNS was same as the ICT, and the duration of each set of tVNS was equal with the duration of each set of ICT, which was approximately 60 min. Except for the different electrode positions, the parameters of tVNS and sham tVNS were consistent.

### Training Session

#### Stop-Signal Task

**FIGURE 2:**
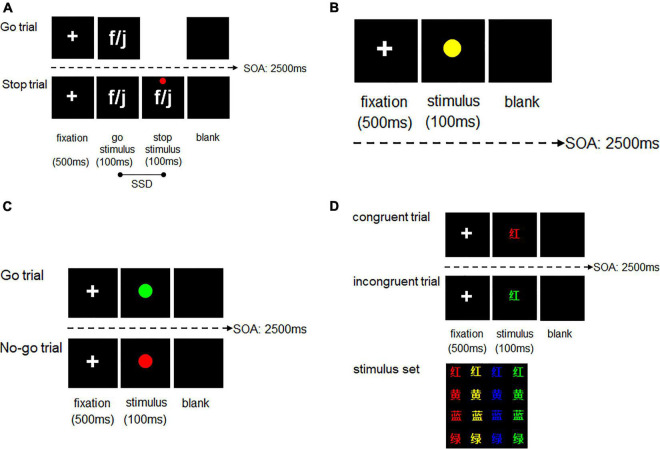
**(A)** SST design in the training session of the current study. **(B)** SRT design in the training session of the current study. **(C)** Go/No-go task design in the pre- and post-training tests of the current study. **(D)** Color-word Stroop task design in the pre- and post-training tests of the current study; “红”, “黄”, “蓝” and “绿” separately represent “red,” “yellow,” “blue,” and “green.”

In the SST, there are a series of go and stop trials. Participants were instructed to use the left or right index finger to press the “f” and “j” keys when go trials appeared, which was the letter f or j presented randomly, and to withhold a response when stop signals appeared, which was a red dot that appeared above the go stimulus letters. Participants were required to respond as quickly and as accurately as possible and not have a delay for the appearance of the stop signal. A white fixation point was presented in the center of a black background for 500 ms, followed by presenting the letter f or j (Figure 2A). The signal of stop trials that was a red cross above the letter of go trials was presented after the time of stop-signal delay (SSD). The SSD was adjusted according to participants’ performance on the stop trials; the initial SSD was 250 ms, and after reacting correctly to a stop trial, the SSD increased by 50 ms, whereas after reacting incorrectly to a stop trial, the SSD decreased by 50 ms. The SSD ranged from 0 to 750 ms. A blank screen was presented for varying durations to ensure that the stimulus onset asynchrony was 2500 ms. Each set of the SST had 240 trials, which was divided into three blocks, and the ratio of go trials to stop trials was 3:1. Participants were permitted to have breaks during the interval of the blocks and the sets of the task. Participants did not begin the test phase until they reached an accuracy level of ≥85% in the practice phrase. Performance on the SST was assessed by stop-signal reaction time (SSRT), which was calculated as the average difference between the reaction time (RT) of go trials and the SSD. A lower SSRT reflected stronger response inhibition. The exclusion criteria in the SST analyses were that it excluded ‘trials’ with too short (less than 150 ms) or too long (more than 1000 ms) RT of go trials or stop-failure trials, excluded “participants” that the mean RT of stop-failure trials longer than the mean RT of go trials and excluded “participants” that p(respond| signal) was lower than 0.25 or higher than 0.75 (Congdon et al., 2012; Verbruggen et al., 2019).

#### Simple Reaction Task

The simple reaction task (SRT) was used as the sham ICT. Participants were presented with go trials only and instructed to use the right index finger to press the down arrow button when go trials of a yellow dot were displayed in the center of the screen. Other parameters of the SRT were the same as those of the SST (Figure 2B).

#### Transcutaneous Vagus Nerve Stimulation

Two Ag/AgCl electrodes (4.5 mm in diameter) of the tVNS stimulator (tVNS501, Rishena, Changzhou, China) were placed at the cymba conche of the left ear to stimulate the auricular branch of the vagus nerve. For sham tVNS, electrodes were placed on the left earlobe. The parts of the left ear that were to be stimulated were cleaned using an alcohol pad, and skin impedance was reduced by applying a gel (Nuprep Gel, Weaver, Colorado, United States) before the stimulation phase. Based on previous tVNS research, stimulation parameters were pulse width of 200–300 μs at 25 Hz and a biphasic pulse interval of 30 s ON and 30 s OFF. The stimulus intensity of the tVNS varied between individuals and was set to the average level, which was defined by the level above detection threshold but below pain perception (Ellrich, 2011; Pihlaja et al., 2020). Because the right auricular vagal nerve projects to the heart, the stimulation was always applied to the left ear to avoid potential side effects to the heart.

#### Pre- and Post-training Tests

Three tasks related to IC were used as pre- and post-training tests, which were carried out in the laboratory and presented using E-prime 3.0 (Psychology Software Tools, Inc., Sharpsburg, PA, United States). The pre- and post-training tests consisted of the SST, the Go/No-go task, and the color-word Stroop (cwStroop) task.

#### Stop-Signal Task

The SST for the pre- and post-training tests was the same as that in the training session and was used to assess the training effect.

#### Go/No-Go Task

The Go/No-go task was used to evaluate the near-transfer effect and is a typical paradigm to assess participants’ response inhibition ability. The task includes two types of picture stimuli: a green circle that indicates a go trial and a red circle that indicates a no-go trial (Figure 2C). The ratio of go trials to no-go trials was 3:1, respectively. Participants were instructed to respond as fast as possible when a stimulus appeared randomly (using the right index finger to press the “j” key when a go trial appeared and withholding a response when a no-go trial appeared). In the Go/No-go task, participants were required to complete the practice phase first and did not enter the test phase until their accuracy rate during the practice reached ≥85%. There were 80 trials in the practice phase and 240 trials in the test practice (180 go trials and 60 no-go trials). The RTs of go trials (go-RT) were used as an indicator of response inhibition.

#### Color-Word Stroop Task

The cwStroop task, which was modified from the Stroop task (Stroop, 1935), was used to investigate the far-transfer effect, which reflected an individual’s conflict inhibition ability. The task included a series of congruent and incongruent trials (congruent word stimuli had the same color and meaning, whereas incongruent word stimuli had differing color and meaning). The word stimuli included one of four Chinese words (“红”, “黄”, “蓝”, “绿”), which are equivalent to the color words of “red,” “yellow,” “blue,” “green.” The color stimuli included one of four colors (red, yellow, blue, and green). Participants were instructed to respond as fast as possible when a stimulus appeared randomly (i.e., using the left index finger to press the “f” key when a congruent trial occurred and using the right index finger to press the “j” key when an incongruent trial occurred; Figure 2D). Participants completed the practice phase first and did not begin the test phase until their accuracy rate during the practice phase reached ≥85%. There were 80 trials in the practice phase and 240 trials in the test phase, which consisted of 120 congruent trials and 120 incongruent trials. The Stroop effect was assessed by the difference in the mean RT between the incongruent and congruent trials. A lower Stroop effect score reflected stronger conflict inhibition.

### Statistical Analyses

Data were analyzed using SPSS 25 (IBM Inc., New York, NY, United States), and the significant level α was set to 0.05. The effect size was estimated using partial eta-squared (ηp^2^). Baseline variables, such as age and simulation intensity, were compared between groups using a one-way analysis of variance (ANOVA). We performed a 4 × 2 repeated-measures ANOVA to analyze the behavioral variables, which included go-RT for the Go/No-go task, SSRT for the SST and Stroop effect for the cwStroop task. We analyzed the main effects of group and session as well as interaction effects. We used simple effects tests to further compare variables between groups and sessions when interaction effects were significant.

## Results

There was no significant difference in mean age between the groups (*F*_(3,54)_ = 0.44, *p* = 0.73, η_p_^2^ = 0.02).

### Performance of the Training Effect

We performed a 4 (group) × 2 (session) ANOVA for behavioral results of the SST (Table 1) to examine the performance of the training effect.

**TABLE 1:** The mean of SST in pre- and post-training tests (M ± SD).

	ICT + tVNS		ICT + sham tVNS		sham ICT + tVNS		sham ICT + sham tVNS		*p*	*F*_(3,54)_
				
	Pre-	Post-	Pre-	Post-	Pre-	Post-	Pre-	Post-		
SSRT	259.47 ± 54.62	197.74 ± 18.01	260.06 ± 28.72	210.52 ± 17.16	241.82 ± 30.82	224.59 ± 30.64	228.39 ± 27.62	213.41 ± 24.76	0.00	8.89
SSD	210.18 ± 97.29	226.73 ± 86.01	245.06 ± 176.72	200.00 ± 75.10	277.05 ± 165.90	258.44 ± 134.58	287.50 ± 110.75	215.00 ± 68.71	0.32	1.19
go RT	469.65 ± 94.08	424.47 ± 76.42	505.12 ± 182.08	410.53 ± 69.36	518.88 ± 149.49	483.04 ± 120.69	515.89 ± 105.84	428.41 ± 62.83	0.49	0.81
p(respond| signal)	48.81 ± 5.00	49.05 ± 4.17	48.11 ± 7.94	50.22 ± 2.26	46.89 ± 6.81	46.67 ± 6.20	47.26 ± 4.65	50.00 ± 2.36	0.47	0.86
Omissions	0.20 ± 0.60	0.12 ± 0.24	0.11 ± 0.23	0.04 ± 0.14	0.07 ± 0.29	0.07 ± 0.29	0.00 ± 0.00	0.04 ± 0.15	0.79	0.35

*M, ms, mean value; SD, ms, standard deviation; p and F_(3,54)_ are the rmANOVA result of interaction effect of group × session; go RT, ms, RT of go trials; p(respond| signal),%, the probability to respond after the stop signal appeared; omissions,%, the probability to miss response after the go trial appeared.*

**FIGURE 3:**
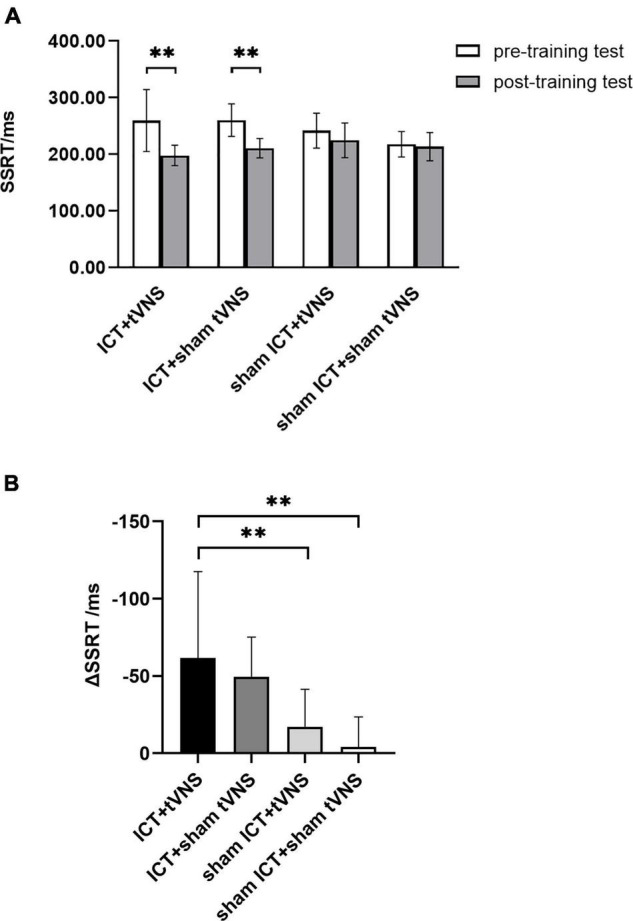
**(A)** The performance of SST tests: SSRT of the SST between pre- and post-training tests. **(B)** The performance of SST tests: The decrement of the SSRT for each group. ^**^P < 0.01, Error bars represent the standard error of the mean.

The results of SSRT revealed a significant main effect of session (Greenhouse–Geisser corrected, *F*_(1,54)_ = 54.84, *p* < 0.01, η_p_^2^ = 0.50) and a significant interaction effect of group × session (Greenhouse–Geisser corrected, *F*_(3,54)_ = 8.89, *p* < 0.01, η_p_^2^ = 0.33). There was no significant main effect of group (*F*_(3,54)_ = 1.78, *p* = 0.16, η_p_^2^ = 0.09). To further explore the training effect, results of the simple effects test (Figure 3A) showed significantly shorter SSRT in the post-training test than in the pre-training test in the tVNS + ICT (SSRT_pre_ = 259.47 ± 54.62, SSRT_post_ = 197.74 ± 18.01, *p* < 0.01) and sham tVNS + ICT (SSRT_pre_ = 260.06 ± 28.72, SSRT_post_ = 210.52 ± 17.16, *p* < 0.01) groups. No significant difference between pre- and post-training tests was found in the tVNS + sham ICT (SSRT_pre_ = 241.82 ± 30.82, SSRT_post_ = 224.59 ± 30.65, *p* = 0.06) or sham tVNS + sham ICT (SSRT_pre_ = 217.62 ± 22.58, SSRT_post_ = 213.41 ± 24.76, *p* = 0.65) groups. The analysis of decrement in SSRT between groups (Figure 3B) using *post hoc* Bonferroni tests showed that there was significant difference in the decrease in SSRT between the ICT + tVNS and sham ICT + tVNS groups of (*p* < 0.01) and between the ICT + tVNS and sham ICT + sham tVNS groups (*p* < 0.01). However, no significant difference was found between the ICT + tVNS and ICT + sham tVNS groups (*p* = 1.00).

The results of SSD revealed that neither the main effect of session (Greenhouse–Geisser corrected, *F*_(1,54)_ = 3.05, *p* = 0.09, η_p_^2^ = 0.05), nor the main effect of group(Greenhouse–Geisser corrected, *F*_(3,54)_ = 0.77, *p* = 0.51, η_p_^2^ = 0.04), nor the interaction effect of group × session (Greenhouse–Geisser corrected, *F*_(3,54)_ = 1.19, *p* = 0.32, η_p_^2^ = 0.06) was significant.

The results of RT of go trials revealed that a significant main effect of session (Greenhouse-Geisser corrected, *F*_(1,54)_ = 15.87, *p* < 0.01, η_p_^2^ = 0.23). There was no significant main effect of group (*F*_(3,54)_ = 0.86, *p* = 0.47, η_p_^2^ = 0.05) or group × session interaction effect (*F*_(3,54)_ = 0.81, *p* = 0.49, η_p_^2^ = 0.04). Furthermore, the analysis of the decrement in RT of go trials between groups using *post hoc* Bonferroni tests revealed no significant differences among the four groups.

The results of p(respond| signal) revealed that neither the main effect of session (Greenhouse–Geisser corrected, *F*_(1,54)_ = 2.49, *p* = 0.12, η_p_^2^ = 0.04), nor the main effect of group(Greenhouse–Geisser corrected, *F*_(3,54)_ = 0.90, *p* = 0.45, η_p_^2^ = 0.05), nor interaction effect of group × session (Greenhouse–Geisser corrected, *F*_(3,54)_ = 0.86, *p* = 0.47, η_p_^2^ = 0.05) was significant.

The results of omissions rate on go trials revealed that neither the main effect of session (Greenhouse–Geisser corrected, *F*_(1,54)_ = 0.34, *p* = 0.56, η_p_^2^ = 0.01), nor the main effect of group(Greenhouse–Geisser corrected, *F*_(3,54)_ = 0.94, *p* = 0.43, η_p_^2^ = 0.05), nor interaction effect of group × session (Greenhouse–Geisser corrected, *F*_(3,54)_ = 0.35, *p* = 0.79, η_p_^2^ = 0.02) was significant.

### Performance of the Near-Transfer Effect

We performed a 4 (group) × 2 (session) ANOVA for behavioral results of the Go/No-go task (Table 2) to analyze the performance of the near-transfer effect.

**TABLE 2:** The mean of Go/No-go in pre- and post-training tests (M ± SD).

	ICT + tVNS		ICT + sham tVNS		sham ICT + tVNS		sham ICT + sham tVNS		*p*	*F*_(3,54)_
				
	Pre-	Post-	Pre-	Post-	Pre-	Post-	Pre-	Post-		
Go-RT	309.60 ± 35.81	275.74 ± 40.19	300.76 ± 41.01	287.12 ± 31.39	275.02 ± 36.63	272.33 ± 27.25	276.20 ± 28.23	278.30 ± 15.63	0.00	5.24
False alarm	10.83 ± 9.00	14.76 ± 11.03	14.11 ± 10.58	17.44 ± 11.68	27.00 ± 19.32	27.78 ± 12.27	20.48 ± 14.18	14.64 ± 8.20	0.08	2.41

*M, ms, mean value; SD, ms, standard deviation; p and F_(3,54)_ are the rmANOVA result of interaction effect of group × session; False alarm, %, the probability to respond after the No-go trial appeared.*

**FIGURE 4:**
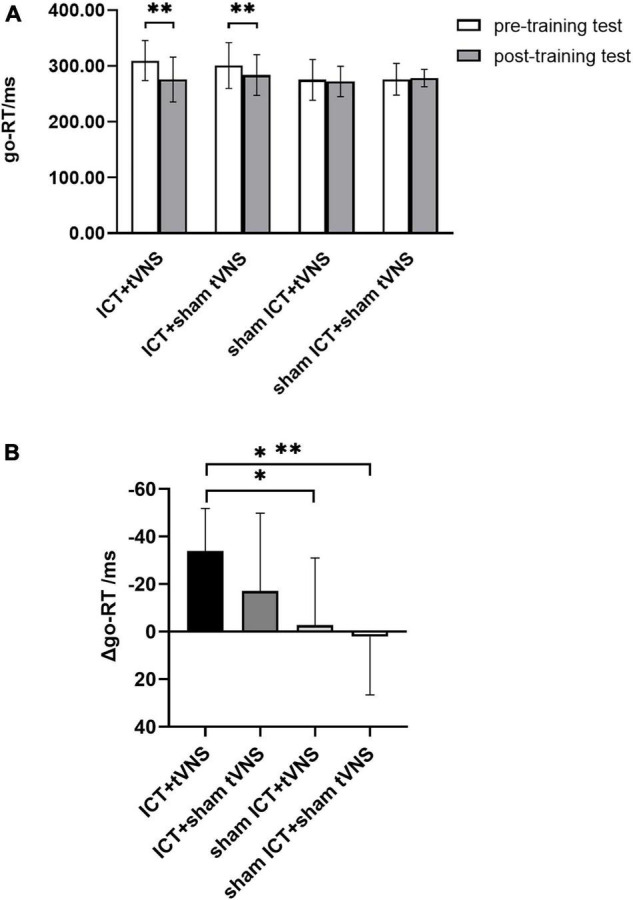
**(A)** The performance of Go/No-go tests: go-RT of the Go/No-go between pre- and post-training tests. **(B)** The performance of Go/No-go tests: The decrement of the go-RT for each group. *P < 0.05, ^**^P < 0.01, Error bars represent the standard error of the mean.

The results of go-RT revealed that a significant main effect of session (Greenhouse–Geisser corrected, *F*_(1,54)_ = 13.57, *p* < 0.01, η_p_^2^ = 0.20) and a significant interaction effect of group × session (Greenhouse–Geisser corrected, *F*_(3,54)_ = 5.24, *p* < 0.01, η_p_^2^ = 0.23). There was no significant main effect of group (*F*_(3,54)_ = 1.50, *p* = 0.23, η_p_^2^ = 0.08). To further explore the training effect, results of the simple effects test (Figure 4A) showed a significantly shorter go-RT in the post-training test than in the pre-training test in the tVNS + ICT (go-RT_pre_ = 309.60 ± 35.81, go-RT_post_ = 275.74 ± 40.19, *p* < 0.05) and sham tVNS + ICT (go-RT_pre_ = 300.76 ± 41.01, go-RT_post_ = 283.73 ± 36.41, *p* < 0.01) group. No significant difference between the pre- and post-training tests was found in the tVNS + sham ICT (go-RT_pre_ = 275.02 ± 36.63, go-RT_post_ = 272.33 ± 27.25, *p* = 0.70) and sham tVNS + sham ICT (go-RT_pre_ = 276.20 ± 28.23, go-RT_post_ = 278.30 ± 15.63, *p* = 0.77). The analysis of the decrement in go-RT between groups (Figure 4B) using *post hoc* Bonferroni tests showed that there was a significant difference in the decrease in go-RT between the ICT + tVNS and sham ICT + tVNS groups (*p* < 0.05) and between the ICT + tVNS and sham ICT + sham tVNS groups (*p* < 0.01). However, no significant difference was found between the ICT + tVNS and ICT + sham tVNS groups (*p* = 0.57).

The results of false alarm rate revealed that neither the main effect of session (Greenhouse–Geisser corrected, *F*_(1,54)_ = 0.15, *p* = 0.70, η_p_^2^ = 0.03), nor interaction effect of group × session (Greenhouse–Geisser corrected, *F*_(3,54)_ = 2.41, *p* = 0.08, η_p_^2^ = 0.12) was significant.

### Performance of the Far-Transfer Effect

**FIGURE 5:**
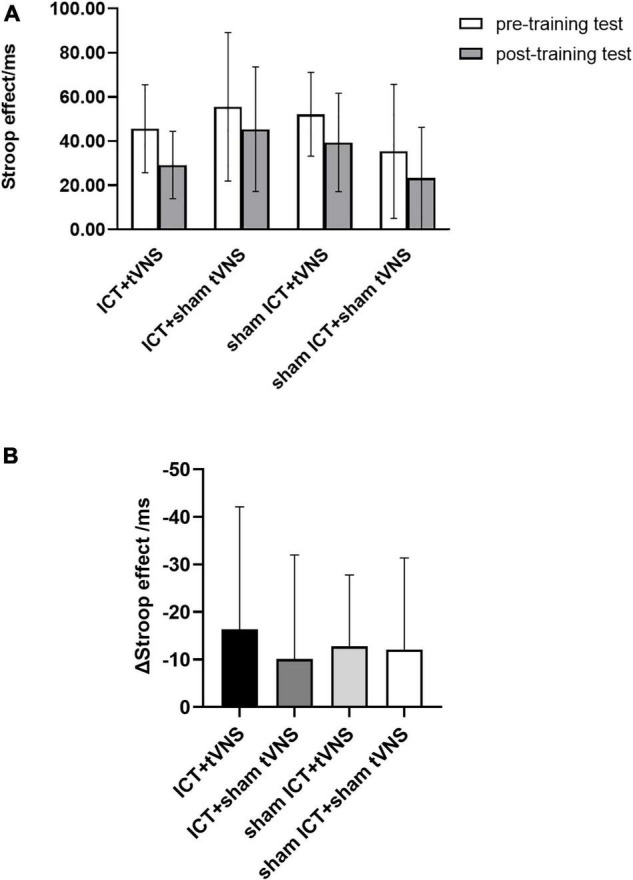
**(A)** The performance of cwStroop tests: Stroop effect of the cwStroop between pre- and post-training tests. **(B)** The performance of cwStroop tests: The decrement of the Stroop effect for each group. Error bars represent the standard error of the mean.

We performed a 4 (group) × 2 (session) ANOVA for the Stroop effect to analyze the performance of the far-transfer effect. Results (Figure 5A and Table 3) revealed a significant main effect of session (Greenhouse-Geisser corrected, *F*_(1,54)_ = 22.14, *p* < 0.01, η_p_^2^ = 0.29). There was no significant main effect of group (*F*_(3,54)_ = 2.50, *p* = 0.07, η_p_^2^ = 0.12) or group × session interaction effect (*F*_(3,54)_ = 0.23, *p* = 0.88, η_p_^2^ = 0.01). Furthermore, the analysis of the decrement in the Stroop effect between groups (Figure 5B) using *post hoc* Bonferroni tests revealed no significant differences among the four groups.

**TABLE 3:** The mean of cwStroop in pre- and post-training tests (M ± SD).

	ICT + tVNS		ICT + sham tVNS		sham ICT + tVNS		sham ICT + sham tVNS		*p*	*F*_(3,54)_
				
	Pre-	Post-	Pre-	Post-	Pre-	Post-	Pre-	Post-		
Stroop effect	45.59 ± 19.91	29.22 ± 15.25	55.53 ± 33.60	45.41 ± 28.23	52.14 ± 18.96	39.39 ± 22.31	35.37 ± 30.31	23.25 ± 23.07	0.88	0.23

*M, ms, mean value; SD, ms, standard deviation.*

## Discussion

The present study aimed to investigate whether a synergistic effect of tVNS and ICT on the improvement of IC exists in healthy adults. We analyzed the training, near-transfer, and far-transfer effects according to the behavioral performance of pre- and post-training tests.

### Training Effect on the Performance of the Stop-Signal Task

The project of targeted cognitive training for improving IC was the SST, which was used to assess the training effect. We found that SSRT of the SST test significantly decreased between the pre- and post-training tests in the ICT + tVNS and ICT + sham tVNS groups, which suggested that participants could obtain the training effect of improving IC by undergoing ICT. Moreover, the decrease in SSRT in the ICT + tVNS group was larger than that in the ICT + sham tVNS group. This indicated that tVNS augments the training effect of ICT, which supports the theoretical mechanism whereby non-invasive brain stimulation augments the training effect *via* targeted cognitive training. The difference in SSRT between the pre- and post-training tests was not significantly different in the sham ICT + tVNS group, which is in line with previous study findings, whereby tVNS alone does not induce a training effect on behavior (Pihlaja et al., 2020).

### Near-Transfer Effect on the Performance of the Go/No-Go Task

The results of the Go/No-go task provided evidence of the near-transfer effect for response inhibition. Go-RT in the Go/No-go task decreased significantly between pre- and post-training tests in the ICT + tVNS and ICT + sham tVNS groups. Moreover, the decrease in go-RT in the ICT + tVNS group was larger than that in the ICT + sham tVNS group. These results showed that tVNS augments the near-transfer effect of ICT, which supports the theoretical mechanism that non-invasive brain stimulation augments transfer effects by *via* targeted cognitive training. Go-RT in the sham ICT + tVNS group did not show a significant decrease between pre- and post-training tests, which indicated that tVNS alone does not have a near-transfer effect on behavior; this is consistent with the previous study on non-invasive brain stimulation for IC improvement (Fischer et al., 2018).

### Far-Transfer Effect on the Performance of the Stroop Test

We adapted the cwStroop task to test the far-transfer effect, which is the improvement in conflict inhibition ability *via* response inhibition training. Although a significant main effect of session was found, there was no significant main effect of group or interaction effect. Results showed that ICT could not induce the far-transfer effect, which is consistent with several previous studies (Kassai et al., 2019; Sala and Gobet, 2019; Zhao and Jia, 2019). Furthermore, combining ICT with tVNS did not induce the far-transfer effect, according to the results of the cwStroop task.

This study suggested that tVNS facilitates ICT in augmenting training and near-transfer effects but not the far-transfer effect. The lack of far-transfer effect is consistent with previous studies suggesting that response inhibition and conflict inhibition refer to separable constructs of IC and the response inhibition tasks may not involve a separate component of IC compared to conflict inhibition tasks, such as cwStroop mainly requires the suppression of an interference response, whereas SST mainly refer to suppress the inappropriate response (Stahl et al., 2014; Zhao and Jia, 2019). Although some previous neuroimaging studies on IC (Aron et al., 2014; Depue et al., 2016; Friehs et al., 2021) found that the neural mechanisms of response inhibition (e.g., SST) and conflict inhibition (e.g., Stroop task) potentially depended on the similar key brain region of the dorsolateral prefrontal cortex (DLPFC), they seemed to closely connect with different types of neural underpinnings on the process of inhibition control. In the SST paradigm of response inhibition, when the DLPFC monitors the situation for the requirement to stop a response and the requirement arises, the DLPFC transfers the signal to the right the inferior frontal gyrus (IFG), which has been assigned a key role as a behavioral “brake” to stop the response (Aron et al., 2014). Meanwhile the IFG will modulate activity in the pre-supplementary motor cortex (pre-SMA) and the motor cortex *via* the subthalamic nucleus to execute stop action (Friehs et al., 2021). Conflict effects of the Stroop task drive the right DLPFC activation, which may contribute to diminish the interference processing from the irrelevant stimulus (Kerns et al., 2004; Egner, 2007; Friehs et al., 2020). Furthermore, the practice effect of cwStroop is sensitive to the anterior cingulate cortex (ACC) activation. The ACC activation effect will weaken with increase number of training sessions while the DLPFC activation is not sensitive to the practice effect of cwStroop, reflecting the top-down attentional control processing (Milham et al., 2003). The differences in neural underpinnings between response inhibition and conflict inhibition provided a reliable reason that no far transfer effects were observed in present study.

The results of the current study suggesting that ICT based on response inhibition tasks did not contribute to the far-transfer effect in conflict inhibition tasks further fits and strengthens the prevailing theory that IC involves multiple separable constructs (Nigg, 2000; Diamond, 2013).

Furthermore, non-invasive brain stimulation is thought to augment intervention effects by modulating neurons and neural networks activated by targeted cognitive training (Elmasry et al., 2015; Mannarelli et al., 2020). These findings may explain why did not observe the far-transfer effect when combining tVNS with ICT. However, the SST and the Go/No-go task involve the same mechanism of response inhibition, which may be why combining ICT with tVNS induced synergistic effects on improving IC. Our finding of training and near-transfer effects supports the theory that combining targeted cognitive training (e.g., ICT) with non-invasive brain stimulation (e.g., tVNS) enhances the effects of cognitive intervention (Elmasry et al., 2015).

The present study suggested that ICT combined with tVNS has a synergistic effect on IC improvement in healthy adults. Thus, ICT combined with tVNS may be a valuable intervention for improving IC in healthy individuals who are in certain industries. Moreover, our findings offer novel research ideas for using tVNS to improve cognition.

This study has several limitations. Firstly, all participants were young undergraduate males, whose baseline level of IC may be too high, which may have caused a ceiling effect. Thus, the results may be incomplete, especially for the investigation of the far-transfer effect. Secondly, we did not include a follow-up measurement to investigate the long-term effect of ICT combined with tVNS. Finally, we did not include electrophysiological indices of IC or explore brain physiology underlying the method of combining ICT with tVNS.

In future studies, we plan to address these limitations by investigating the neuromodulatory effects of tVNS on ICT and obtaining 3- or 6-month follow-up measurements of beneficial transfer effects. In addition, we plan to recruit individuals of different sex, age, and educational background to investigate the effects of individual differences on the combination of ICT and tVNS.

## Data Availability Statement

The raw data supporting the conclusions of this article will be made available by the authors, without undue reservation.

## Ethics Statement

The studies involving human participants were reviewed and approved by the Medical Ethics Committee of the Air Force Medical University. The patients/participants provided their written informed consent to participate in this study. Written informed consent was obtained from the individual(s) for the publication of any potentially identifiable images or data included in this article.

## Author Contributions

CW and ZW designed and conducted the study. XC contributed to the study design. CW, ZG, and YL contributed to recruitment of participants, data collection, and data analysis. CW was responsible for writing of the manuscript. All authors approved the final manuscript and contributed to the article and approved the submitted version.

## Conflict of Interest

The authors declare that the research was conducted in the absence of any commercial or financial relationships that could be construed as a potential conflict of interest.

## Publisher’s Note

All claims expressed in this article are solely those of the authors and do not necessarily represent those of their affiliated organizations, or those of the publisher, the editors and the reviewers. Any product that may be evaluated in this article, or claim that may be made by its manufacturer, is not guaranteed or endorsed by the publisher.
